# Conducting a meta-ethnography of qualitative literature: Lessons learnt

**DOI:** 10.1186/1471-2288-8-21

**Published:** 2008-04-16

**Authors:** Salla Atkins, Simon Lewin, Helen Smith, Mark Engel, Atle Fretheim, Jimmy Volmink

**Affiliations:** 1 Health Systems Research Unit, Medical Research Council of South Africa, Cape Town, South Africa; 2 South African Cochrane Centre, Medical Research Council of South Africa, Cape Town, South Africa; 3 Primary Health Care Directorate, University of Cape Town, Cape Town, South Africa; 4 Department of Public Health and Policy, London School of Hygiene and Tropical Medicine, London, UK; 5 International Health Group, Liverpool School of Tropical Medicine, UK; 6 Department of Medicine, University of Cape Town, Cape Town, South Africa; 7 Norwegian Knowledge Centre for the Health Services, Norway; 8 University of Stellenbosch, Cape Town, South Africa

## Abstract

**Background:**

Qualitative synthesis has become more commonplace in recent years. Meta-ethnography is one of several methods for synthesising qualitative research and is being used increasingly within health care research. However, many aspects of the steps in the process remain ill-defined.

**Discussion:**

We utilized the seven stages of the synthesis process to synthesise qualitative research on adherence to tuberculosis treatment. In this paper we discuss the methodological and practical challenges faced; of particular note are the methods used in our synthesis, the additional steps that we found useful in clarifying the process, and the key methodological challenges encountered in implementing the meta-ethnographic approach.

The challenges included shaping an appropriate question for the synthesis; identifying relevant studies; assessing the quality of the studies; and synthesising findings across a very large number of primary studies from different contexts and research traditions. We offer suggestions that may assist in undertaking meta-ethnographies in the future.

**Summary:**

Meta-ethnography is a useful method for synthesising qualitative research and for developing models that interpret findings across multiple studies. Despite its growing use in health research, further research is needed to address the wide range of methodological and epistemological questions raised by the approach.

## Background

Qualitative research has become more commonplace within health services research [1-3]. The increasinolume of qualitative research available has drawn attention to synthesis as one means of combining knowledge gathered from individual studies and of developing theory [4]. Much of the groundwork in developing methods for synthesizing the findings of qualitative studies has been conducted in the health and education fields. Within health care, these developments have been led, to some extent, by the growth of systematic reviewing as a tool for synthesizing evidence on the effectiveness of health care interventions. These efforts have highlighted both the limitations of systematic reviews of effectiveness in explaining the effects of interventions [5] and raised interest in synthesis in the interpretive paradigm.

Qualitative research is concerned primarily with how people see and understand their social worlds [6]. Qualitative approaches may offer explanations for unexpected or anomalous findings emerging from quantitative research and may also elucidate relationships identified in these studies [7]. Assembling the findings of multiple primary qualitative studies using a systematic process may have a number of additional benefits: they may help generate more comprehensive and generalisable theory; they may add greater breadth and depth to existing systematic reviews of effectiveness by focusing on the views of those towards whom the interventions are directed [8]; or they may provide insights into the reasons why interventions succeed or fail [9]. In doing so, reviews of qualitative studies may usefully inform the implementation of interventions and programmes.

Although combining the findings of studies using qualitative approaches appears to be a worthwhile exercise, the nature of qualitative research raises challenges for its evaluation and synthesis [10]. Key issues include the differing philosophical assumptions underpinning studies within the interpretivist paradigm, such as those drawing on phenomenological or ethnographic approaches, and whether or how to synthesize the findings of such studies. Concerns have also been expressed regarding the loss of explanatory context when the findings of multiple studies are combined, particularly given the importance of context in the analysis and interpretation of qualitative data. Whether and how to critically appraise qualitative studies included in a synthesis is also problematic. Some authors suggest that this imposes a positivist approach to 'quality' on studies conducted within a very different tradition [11].

A number of approaches to the synthesis of qualitative data have been proposed. Some are based on analysis methods used in primary research and most represent either an integrative or interpretive approach to synthesis [5,7]. Meta-ethnography is an interpretive approach originally developed by Noblit and Hare for combining the findings of ethnographic research conducted in the field of education [12]. This synthesis method has the potential to provide a higher level of analysis, generate new research questions and reduce duplication of research. The approach has been used for research syntheses in health care, particularly for questions relevant to patient experiences of illness and care, such as lay experiences of chronic illness, of which there are now a number of published examples [4,13,14]. Some authors suggest that the strength of this approach lies in its attempt to preserve the interpretive properties of primary data [15], but there are several methodological questions surrounding its use for combining primary research, particularly research from different theoretical perspectives.

Our qualitative review was prompted by a Cochrane systematic review of randomised controlled trials of directly observed therapy (DOT) versus self-administered treatment for improving adherence to tuberculosis (TB) treatment. This review showed no quantitatively important effect of DOT on cure or treatment completion in people receiving treatment for TB [16]. Despite the wide implementation of DOT, poor adherence to treatment remains a key reason for the failure to contain the epidemic in many contexts and, in response to this, a large number of qualitative studies of this problem have been conducted. Therefore we decided to seek out qualitative research exploring patient experiences of taking TB treatment, in an attempt to identify the types of factors that could influence treatment adherence. In other words, we sought other evidence, beyond reports of the effects of one intervention, which could help explain treatment adherence behaviour. Systematically examining the body of qualitative evidence could also help inform policy and practice, including the design of more appropriate and effective interventions to promote adherence to TB therapy.

In this paper we discuss the challenges of applying a meta-ethnographic approach to synthesising qualitative research on TB medication adherence. The findings of the synthesis are reported elsewhere [17] and summarised in Table 1. We report here on our interpretation of each of the seven steps outlined by Noblit and Hare in the original description of meta-ethnography [12]. For each step, we describe the problems that we encountered and areas for further methodological research.

**Table 1:** Description of our meta-ethnography

**Experiences of tuberculosis treatment: A meta-ethnographic analysis of facilitators and barriers to inform the development of an adherence-promoting intervention**	
Aim	To determine barriers and facilitators of tuberculosis adherence

Search strategy	Text words included: Tuberculosis, adherence, compliance and concordance; obtained 7814 abstracts

Quality assessment	Assessed for quality using 13 criteria, extracted data

Synthesis approach	Meta-ethnographic analysis of 44 primary studies using both reciprocal translation and line of argument synthesis to develop a third order interpretation.

Key findings	A model that indicated adherence is related to: structural factors, social influence, organisation of treatment and care, and factors related to the disease

Hypotheses emerging from the synthesis	Increasing the visibility of TB programmes in the community may increase knowledge and improve attitudes towards TB Providing more information about the disease and treatment to patients and communities Increased support from family, peers and social networks Minimizing costs and unpleasantness related to clinic visits and increasing flexibility and patient autonomy Increasing flexibility in terms of patient choice of treatment plan and type of support Increasing the patient centredness of interactions between providers and clients Addressing 'structural' and 'personal' factors, for example through micro-financing and other empowerment initiatives Providing more information about the effects of medication to reduce the risk of patients becoming non-adherent when experiencing treatment side effects

## Discussion

### Step 1: Getting started

According to the original method [12], 'getting started' involves determining a research question that could be informed by qualitative research. In our case, the equivocal evidence from the original systematic review of strategies to improve adherence to TB treatment [16] provided the rationale for approaching the issue of adherence from the perspective of patients and other stakeholders. A further motivation for synthesizing the body of qualitative evidence was to inform the development of patient-oriented interventions to improve adherence rates to TB treatment in South Africa. We felt that the opinions, attitudes and knowledge of patients regarding treatment, and a theoretical model of adherence behaviour could shape future interventions to promote adherence. This step was fairly straightforward to execute and the team found it relatively easy to decide on the focus of the synthesis, and the contribution it would make to current debates on adherence to TB treatment regimens.

### Step 2: Deciding what is relevant to the initial interest

The second step in the process – 'deciding what is relevant to the initial interest' – appeared to involve several distinct decisions and processes. Based on our experience, we suggest that these are: defining the focus of the synthesis; locating relevant studies; making decisions on inclusion; and quality assessment.

#### Defining the focus of the synthesis

An important first decision was whether to include all studies discussing experiences of TB treatment; we needed to find a balance between a broad scope review, and a focus that would yield a manageable number of studies. We chose to focus on qualitative findings that would inform the design of interventions to improve adherence to TB treatment across a range of settings, and limited the inclusion criteria to studies that clearly used known qualitative research methods, addressed adherence to curative or prophylactic TB treatment, and described adherence from the perspective of patients, caregivers or other stakeholders. We recognise that focussing only on adherence, and not including papers examining the experience of TB more widely, may have resulted in some important papers being overlooked. However, we made this choice in order to ensure a manageable number of papers.

#### Locating relevant studies

The second important component of 'deciding what is relevant to the initial interest' involved locating potentially relevant studies. The indexing and archiving of qualitative research has advanced since meta-ethnography was first proposed, but locating qualitative health research remains a challenge. We conducted free text searches on the topic, using the keywords: "tuberculosis AND (adherence OR concordance OR compliance)". We used free-text searching because we experienced a number of problems in using qualitative research terms as Medical Subject Heading (MeSH) or filter terms. Qualitative research is frequently published in books [18] or theses, and it may also be catalogued in electronic databases outside of the medical domain [19]. For these reasons reliance on Medline alone is discouraged [10]. In developing our search strategy we targeted a large number of databases, including some, such as PsycInfo and the Social Science Citation Index, that are not exclusively medical in focus (Table 2). A further challenge is the descriptive titles used by many qualitative researchers. These could lead to inappropriate indexing of these studies and may also hamper the use of relevant keywords [20] or identification of study design [1]. These issues may result in implicit sampling in the search process, where relevant articles may be inadvertently left out. We supplemented our database searches by citation searching in retrieved papers and by consulting experts to locate studies in progress or known theses on the topic. However, only three of the total of 44 included studies were located in this way.

**Table 2:** Search results

**Database**	**Search dates**	**Total hits**
PsycINFO	1972-	53
ERIC	1966-	6
Academic Search Premier	1965-	205
Health Source: Nursing/Academic	1985-	141
ScienceDirect	1964-	149
Social Science full text	1983-	29
Social science citation expanded, social science citation index, arts and humanities citation index	1975-	889
Medline	1966-	1772
CINAHL	1982-	321
Pre-CINAHL	Current	1
Dissertation abstracts	189--	57
Sociological abstracts, social services abstracts, PAIS international	1963-, 1972-, 1980-	17
EMBASE	1966-	2349
PapersFirst	1993-	12
Pubmed	1966-	1813
**Total**		7814

We were also aware that our reliance on free text words in our search strategy such as 'adherence' or 'compliance' may have restricted the retrieval of relevant studies, such as those providing a more holistic and general view of patient experiences of TB. This, in turn, may have had implications for the review findings, as adherence depends on a variety of contextual factors and is embedded in the general experience of having TB. We acknowledge this limitation, but feel that our narrower focus helped to limit the studies to a more manageable number. Choosing an appropriate approach to locating relevant studies is therefore not straightforward.

#### Inclusion decisions

Systematic reviews of trials attempt to locate every possible study on a given topic or intervention and some authors [18,21] advocate a similar approach for qualitative syntheses. In contrast, and in keeping with the methods of primary qualitative research, other methodologists suggest the use of theoretical sampling until data saturation is reached [22]. Key difficulties with this approach include how to establish the population of studies from which to sample without first identifying all relevant studies. It is also unclear how data saturation is determined in a synthesis, where access to the original data is limited, and little guidance on this is available [7]. Although our search strategy was broad, important research or subgroups may still have been overlooked due to the poor indexing of qualitative research.

We initially selected studies for inclusion based on title scanning, from which papers not meeting our criteria would be filtered out by reading abstracts [21]. However, we found that most papers included poorly structured abstracts or no abstract at all – a common problem encountered by others [20,23]. The uninformative content of abstracts, combined with poor and inconsistent structuring such as lack of description of methods used, made it difficult to make inclusion decisions based on abstracts alone. In order not to exclude potentially eligible studies, we had to scan a very large number of papers for which the abstracts did not allow a definitive decision to be made. In total, over 600 full text articles were reviewed. When applying the review inclusion criteria we also had to decide whether to include studies reporting mixed method research with a qualitative component in addition to those reporting only qualitative research.

Determining whether a paper had used qualitative methods required considerable discussion at times. We decided on an inclusive policy to avoid omitting research of potential value to the synthesis. In practice, however, we found it difficult to apply our inclusion criteria to mixed method studies because of inadequately described methods. All inclusion decisions were based on the consensus of two reviewers.

#### Quality assessment of included studies

Critical appraisal is an important component of systematic reviews of experimental studies, preventing inclusion of poorly conducted trials where there is likely to be bias. The application of quality criteria to qualitative research is widely debated, and currently there is no consensus on whether criteria should be applied, which criteria to use, and how to apply them [2,24]. Authors of published meta-ethnographies are divided on the merits of quality assessment and whether it should form part of a meta-ethnography at all. We chose to assess the quality of included papers in order to explore the contribution of such assessments to the synthesis and also to describe the range of quality found in the papers and major gaps in the reporting of the included research.

We performed a cursory review of several quality criteria [2,15,25-27] and then developed an adapted version of the CASP quality assessment tool [27] to review components which appeared important for our purpose (see Table 3). While other authors have found quality assessment a useful screening process [13], and have eliminated poor quality studies, we made an a priori decision that every paper meeting our basic criteria would be included in the final analysis. We were concerned that the over-rigorous application of the criteria could be counterproductive [13], as the criteria had not been previously tested for their accuracy in identifying good quality studies, and papers that were intuitively good research may not have fared well in the quality assessment. We therefore did not exclude any studies on the basis of the quality assessment.

**Table 3:** Quality criteria and results

**Question**	**Yes**	**No**	**Unclear**
1. Is this study qualitative research?	**43**	**0**	**1**
2. Are the research questions clearly stated?	**38**	**2**	**4**
3. Is the qualitative approach clearly justified?	**13**	**22**	**9**
4. Is the approach appropriate for the research question?	**42**	**2**	
5. Is the study context clearly described?	**24**	**3**	**17**
6. Is the role of the researcher clearly described?	**12**	**27**	**5**
7. Is the sampling method clearly described?	**21**	**11**	**12**
8. Is the sampling strategy appropriate for the research question?	**21**	**6**	**17**
9. Is the method of data collection clearly described?	**31**	**1**	**12**
10. Is the data collection method appropriate to the research question?	**37**	**0**	**7**
11. Is the method of analysis clearly described?	**12**	**20**	**12**
12. Is the analysis appropriate for the research question?	**17**	**10**	**17**
13. Are the claims made supported by sufficient evidence?	**23**	**9**	**12**

We found that appraising the studies became an exercise in judging the quality of the written report rather than the research procedure itself [28]. Papers published in qualitative-oriented journals were easier to evaluate because the length of articles allowed authors to elaborate on the research process [1,13]; more concise papers published in medical journals and mixed-method studies seemed to fare worst in our quality assessment. We also found that papers appearing to have face validity, and that we intuitively felt to be well conducted research, did not necessarily come across as such in our quality assessment.

Through our critical appraisal exercise we identified several methodological aspects that authors consistently failed to report. Only ten papers reported the qualitative approach used in the study: five were ethnographies, three used grounded theory approaches, one noted that they used a "sociological study" and one used a critical theory/constructivist approach. Applied qualitative research studies on adherence, conducted with a priori policy relevant questions in mind, were more likely to use a deductive approach. In this synthesis, the theoretical standpoint of the research team appeared to impact on the reporting of the results. However, it is difficult to discern the impacts that the theoretical orientation of the researchers had on the analysis process itself, and how the findings might have been presented differently if another theoretical framework had been adopted. This raises the question of whether papers from different theoretical perspectives should be synthesized, as it is likely that these different approaches impact on both the framing of the research question and the interpretation of data. Furthermore, these approaches have different standpoints on the nature of data and the position of the researcher. Further methodological research on this issue is clearly needed.

Our assessment also highlighted that most papers failed to describe a recognised approach to analysis, although many appeared to use a thematic approach. We acknowledge that, while rigorous application of methods make for rigorous qualitative research, the two are not entirely interdependent [10], and that poor reporting of methods does not equate with poorly conducted research. We therefore decided that studies that did not include a clear description of the analysis methods might still make a valuable contribution to the synthesis.

It became apparent, though, that study quality had an effect on the contribution of a paper to the overall synthesis. Papers that provided mainly descriptive data offered few insights, while others that included thick description and rigorous analysis contributed more substantively to the themes. We acknowledge that assessments of study quality may not be essential and that the methodological shortcomings of a study can emerge during synthesis. However, assessing quality may draw reviewers' attention to pitfalls in the interpretation of study findings that may have a bearing on the results of the synthesis [29].

### Step 3: Reading the studies

We interpreted Noblit and Hare's next step, "reading the studies", as becoming as familiar as possible with the content and detail of the included studies and beginning the process of extracting 'metaphors' or emerging themes. We extracted data from each of the 44 studies using a standard form to summarise the main themes as well as information on the methods, quality, ethical procedures and the contexts of the research. One researcher extracted data from all studies, and data were extracted from a selection of studies by four additional researchers. This process revealed no important differences in the data extracted; it seemed that the team understood in similar ways the key themes in the included papers. The main differences centred on the selection and length of quotations extracted from the original paper. We chose to record themes and important details as summaries since verbatim extraction would be time consuming for 44 papers, but acknowledge that in summarising we risked losing important detail. We found the process of extracting themes complicated; the focus of studies varied from a patient's general experience of TB as an illness to their experience of adherence to a regimen of directly observed therapy. To guide us, we constantly kept in mind the aims of the synthesis, and returned to the original papers frequently to clarify the context of extracted themes.

Many published examples of meta-ethnographies make use of Schutz's [30] notion of first-, second- and third-order constructs and we attempted to utilise these in the analysis process (see Table 4). However, accessing first order constructs, or participant views or beliefs, is problematic in the context of a meta-ethnography since the data extracts included in the primary papers have already been selected from the full dataset by the study authors. These extracts therefore do not reflect the totality of participant experiences. Author, or second order, interpretations, can provide more insight by offering an explanation of the observed phenomena, but we found that the level of interpretation offered in the papers was minimal – many of the papers were highly descriptive in nature. Furthermore, it was often difficult to distinguish first- from second-order interpretations or to decipher to what extent the authors' interpretations were influenced by their own background or theoretical standpoint. Additional problems were encountered in mixed method papers where the distinction between quantitative and qualitative findings was not always apparent. For these reasons, and because all reported data are the product of author interpretation, the usefulness of Schutz' categorization in undertaking a meta-ethnography remains unclear.

**Table 4:** Definitions

Term	Definition
1^st ^order construct	Constructs that reflect participants' understandings, as reported in the included studies (usually found in the results section of an article).
2^nd ^order construct	Interpretations of participants' understandings made by authors of these studies (and usually found in the discussion and conclusion section of an article).
3^rd ^order construct	The synthesis of both first and second order constructs into a new model or theory about a phenomenon
Reciprocal translation	The comparison of themes across papers and an attempt to "match" themes from one paper with themes from another, ensuring that a key theme captures similar themes from different papers
Line of argument synthesis	The development of a new model, theory or understanding by synthesising and interpreting first and second order themes found in the text.

### Step 4: Determining how the studies are related

In determining how the studies are related, Noblit and Hare [12] suggest creating a list of themes or metaphors, juxtaposing them and determining how they are related; other published meta-ethnographies report using grids or tables to display concepts and themes across all studies. We decided to take the latter approach, but with an emphasis on reducing the themes into relevant categories as we progressed.

Given the relatively large number of papers included in our synthesis (n = 44), we set ourselves some organising principles at this stage. Our original attempt of translating one study to another seemed impossible, as the themes emerging from the studies varied considerably and consecutive studies had few themes or interpretations in common. We therefore used a thematic analysis of themes identified in step 3 to identify nine categories, closely mimicking Pound et al [14]. These categories included, for example, "social factors", "disease progress" and "financial burden", and the data within each category formed the basis for the reciprocal translation described below. As the categories were created on the basis of primary data rather than prior knowledge, we felt that this approach was true to the meta-ethnographic method. We revised and merged these categories (for example, disease progress became a more encompassing category of "interpretations of illness and wellness") through discussion of how they were related and by reference to the original text.

### Step 5: Translating studies into one another

The original method implies comparing the metaphors and concepts in one account with the metaphors and concepts in others [12]; but it was unclear to us exactly how to do this. We decided that the included studies were sufficiently similar in their focus to allow reciprocal translation, but at that stage it was unclear if they built a 'line of argument' (see Table 4 for definitions). We approached the reciprocal translation by first arranging each paper chronologically, thereafter comparing the themes and concepts from paper 1 with paper 2, and the synthesis of these two papers with paper 3, and so on, beginning from our categories created above, but keeping an open mind for emerging ones.

The order in which studies are compared may influence the resulting synthesis, and some argue that starting with an index or 'classic' paper identified by experts in the field may be the best approach [13]. We considered a chronological comparison more appropriate in this case because our included studies ranged over 20 years during which significant shifts in the management of the disease occurred, including the global implementation of the DOTS strategy with its directly observed therapy component. However, the chronological approach was less useful than we anticipated as TB policies, such as the DOTS strategy, were adopted at different times in different countries and also within countries. It was often difficult to ascertain, from the included studies, when these policy changes had taken place and therefore to discern the impacts of these policies, if any. The impacts of chronology on the synthesis therefore appeared to be small. Nonetheless, it is possible that unreported policy changes in the study settings over time might explain some of the differences in findings across papers included in the review.

For each study, we examined in detail the issues related to a given theme – for example, family, community and social support issues, and issues related to organisation of care. As we compared the studies, our initial broad grouping of themes was gradually refined by merging and collapsing categories. While this approach is pragmatic, and assisted in the synthesis of many disparate studies, it is possible that this prior grouping had some effect on the results of the synthesis, and may also have constrained the emergence of new categories. We tried to address this, in part, by ensuring that two reviewers were involved in the translation process. Some new sub-categories, but no new major categories, emerged through this translation. In addition, we found that second order constructs tended to be more complex, and were difficult to translate into one another in a transparent manner.

As context is important in primary qualitative research, and lends credibility and weight to primary studies, the intention of a synthesis is to retain the rich context of the data. We attempted to explore systematically the influence of various contextual factors, such as the socio-economic status of the study populations and their geographic location, on the findings of our synthesis. This was difficult, however, due to poor reporting of contextual information in many studies, possibly due to journal word limits. This has been one of the main critiques of meta-ethnography (Estabrooks et al 1994, cited in [7]). The problem of how to retain the rich context of the primary studies when conducting a synthesis is therefore complicated by the failure of many primary study authors to provide adequate descriptions of context or of the impact of context on findings. Some syntheses have attempted to circumvent this problem by first examining only studies undertaken in a particular context [31], but this remains an important methodological constraint for qualitative synthesis approaches.

### Step 6: Synthesising translations

In the same way a primary study might move from descriptive to explanatory analysis [32], a meta-ethnography can proceed from reciprocal translation to a higher order interpretation which distils the translations into more than the parts alone imply – a "line of argument" synthesis. In a brief review of other published meta-ethnographies, we found that authors had arrived at synthesised translations in a variety of ways. In some examples, third order interpretations seemed to be derived from first and second order constructs reported in the primary studies [4,14,33], but in most cases the process for doing this was not clearly defined. There appears to be a general acceptance that the synthesis process, not unlike analysis in primary qualitative research, "cannot be reduced to mechanistic tasks" [4] and may, in practice, be difficult to replicate. Differences in synthesis approaches may also be due to differences in the extent to which included studies report second order interpretations and in the number of papers included in the synthesis.

As the process of synthesising research in meta-ethnography is not clearly delineated, we agreed on a method of synthesis based on our reading of a number of existing reviews. In developing an overarching model (or third order interpretation or synthesis), we listed the translated themes and subthemes in a table, juxtaposed with secondary themes derived from author interpretations. Each member of the (multi-disciplinary) research team then independently developed an overarching model that linked together the translations and authors' interpretations. These models were then merged, discussed, and used to generate hypotheses, in order to produce a 'line-of-argument' synthesis. Each author was also asked to develop a mind map of their own model of the synthesis. Synthesising results in this manner proved rather difficult, as the interpretations of different members of the team varied widely. Inevitably, compromises needed to be made. This highlights the similarity of qualitative synthesis with primary qualitative research, in terms of the inherent subjectivity of interpretation. We also found that synthesising the large number of studies from many different contexts complicated the synthesis process. Our methods are one way of proceeding, but further examination of synthesis methods is warranted.

### Step 7: Expressing the synthesis

The hypotheses generated by the synthesis on how treatment adherence might be improved, and the model of factors influencing adherence, were important outcomes of this secondary research. In order to make the results easily accessible to a wide audience, we presented both the hypotheses and the model in a simple diagrammatic manner. However, we found that simplifying the complex interactions in adherence behaviour, while necessary for the expression of these results, is not a simple process and may further reduce the influence of context on the results.

Another issue to consider in expressing the synthesis is the uptake of these results into policy, programme development and research. We have published the findings of this synthesis in a leading medical journal and communicated the key points at relevant conferences. The model and hypotheses hopefully convey the main findings of the review to policy makers, practitioners and programme planners, who can use them to develop new interventions to promote adherence. However, how to present these results in a form that is accessible to policy makers, and how to translate these findings into operationalised research questions or interventions to improve treatment adherence, needs further exploration. One of the ways in which these results could contribute further to policy, programmes and research would be through linking the results to the existing Cochrane reviews on the effectiveness of interventions to promote adherence to TB treatment. However, our synthesis had a wider scope than the reviews, and therefore is not directly comparable to these. One of the ways in which this work has been taken forward is by mapping the barriers to tuberculosis treatment adherence, identified by the synthesis, onto known interventions to promote adherence to treatment [34]. More work, however, needs to be conducted on approaches to merging the findings of qualitative and quantitative systematic reviews that address linked questions.

## Conclusion

Using the meta-ethnographic approach demands consideration of the suitability of the method and the rationale for using it. We believe it is important for the review question to drive the synthesis method and that meta-ethnography is probably best-suited to generating models or higher order theories of behaviour or experiences. The most important step in the process is formulating a focused question that will set the boundaries for the scope and depth of the synthesis.

We found having a multidisciplinary team useful, particularly in developing the focus for the synthesis, interpreting the steps of meta-ethnography, and in translating and interpreting meaning in the results of the synthesis. The members of the team had both clinical and social science backgrounds, which brought different perspectives to the review, ensured rigour and transparency in methods and helped the results of the review to be more relevant to planners developing treatment policies. One of the reviewers was an author of the original Cochrane Review on treatment adherence to tuberculosis medication [35], which ensured that we remained focussed on addressing the issues identified in the review. We communicated regularly as a team, by email and teleconference; this helped to clarify procedures and enabled us to document various challenges faced in the process of meta-ethnography.

In using the meta-ethnographic method, we identified aspects of the methodology that require clarification, probably through methodological research but also through careful scrutiny by those applying the method. These include the need to assess the effects of critical appraisal of studies and quality assessment, and exclusions based on these, on the outcomes of the synthesis; how best to approach reciprocal translation, and the order in which to compare studies; how context can best be incorporated into meta-ethnographies; and the effect of synthesis across studies undertaken with different philosophical traditions in qualitative research. The method also fails to offer a robust guide to sampling studies and selecting studies for inclusion. We developed our own inclusion criteria and adhered to an inclusive approach in selecting studies for the synthesis, but it is not clear what effects these decisions may have had on the results of the synthesis. For example, we found that papers included in the review from medical journals tended to fare worse in quality assessment. However, this may not be a reflection on the studies themselves but rather an indication that the process of quality assessment needs much more consideration. It is also unclear how to deal with translating studies into one another when large numbers of studies are included. Our data-driven categories may be one useful approach, and computer-assisted coding and extraction of themes may be another way managing large datasets.

The difficulties that we encountered in searching for qualitative studies are likely to be experienced by all researchers developing search strategies for syntheses that include qualitative evidence. More informative abstracts would greatly assist this process and we encourage editors of all journals that publish qualitative research to insist on structured abstracts that include clear descriptions of: the aim of the research; the methods used, including data collection and analysis procedures; and the findings, including key themes where applicable. Keywords that relate to the methods employed would also be helpful in improving indexing.

The similarity of qualitative synthesis to primary qualitative research means that the outcome of the review is heavily influenced by the reviewers. The reproducibility of the review is therefore an area which could be examined further. Other reviews on treatment adherence have been published recently [14,36], both of which identified similar issues to the issues in our review. The focus of one of the reviews [36] is similar to ours, which offers an opportunity to compare commonality in themes, the identification of relevant articles, and the ways on which third order interpretations differ and the reasons for this. Comparing the results of one synthesis to other syntheses on the same or similar topics may offer a way to validate results.

It is still to be determined whether a meta-ethnography contributes more to literature synthesis than a traditional narrative review would. The method can certainly help arrive at higher order interpretations and generate theory from multiple studies and also usually provides more information on the methods of the review than is the case in traditional narrative reviews.

Meta-ethnography was first developed for combining meaning across ethnographies (primary research that aims to provide an account of a particular community or phenomenon, through thick description of behaviours and practices, and to contribute to theoretical understanding of these social phenomena). These types of study lend themselves well to the generation of third order interpretations because of their thick description of phenomena and their focus on meaning. In contrast, qualitative research in public health is often more applied, and concerned with solutions to problems, evaluation, and policy relevant questions to assist management decisions [37]. Our experience suggests that this research often lacks thick description or even interpretation beyond basic description. In syntheses of mainly descriptive research, third order interpretations may be more dependent on the themes identified in studies than interpretations. However, our process resulted in a third order interpretation that resonated with a number of international public health practitioners working in the TB field, suggesting that the model emerging from these rather descriptive studies may be useful in future research and practice.

Using the meta-ethnographic approach, we were able to produce a model of adherence to TB treatment by re-interpreting meaning across many individual qualitative studies. We also derived plausible hypotheses that can be used by policy makers and programme managers to re-organise treatment and care systems to improve adherence. Adapting the method for use in synthesising qualitative health research raises a number of methodological challenges that require further exploration.

## Summary

Meta-ethnography helps re-interpret meaning across many qualitative studies

A clearly formulated question helps to set the boundaries for the scope and depth of a meta-ethnography

Conducting a meta-ethnography of qualitative health research poses a number of methodological challenges, including locating studies, synthesising these and presenting findings.

Further research should be directed at developing and evaluating methods for synthesising qualitative research.

## Abbreviations

DOT = Directly Observed Therapy; TB = Tuberculosis

## Competing interests

The author(s) declare that they have no competing interests.

## Authors' contributions

SA compiled the main text with writing input from SL and HS. All authors participated equally in the conceptual development and editing of this article.

## Pre-publication history

The pre-publication history for this paper can be accessed here:



## References

[B1] Lloyd Jones M (2004). Application of systematic review methods to qualitative research: practical issues. Journal of Advanced Nursing.

[B2] Mays N (2000). Qualitative research in health care: Assessing quality in qualitative research. British Medical Journal.

[B3] McEwan MJ (2004). A systematic review of the contribution of qualitative research to the study of quality of life in children and adolescents with epilepsy. Seizure.

[B4] Britten N, Campbell R, Pope C, Donovan J, Morgan M, Pill R (2002). Using meta ethnography to synthesise qualitative research: a worked example. J Health Serv Res Policy.

[B5] Mays N, Pope C, Popay J (2005). Systematically reviewing qualitative and quantitative evidence to inform management and policy-making in the health field. J Health Serv Res Policy.

[B6] Green J (2004). Qualitative methods for health research..

[B7] Dixon-Woods M, Agarwal S, Young B, Jones D, Sutton A (2004). Integrative approaches to qualitative and quantitative evidence..

[B8] Thomas J (2004). Integrating qualitative research with trials in systematic reviews.. British Medical Journal.

[B9] Harden A, Garcia J, Oliver S, Rees R, Shepherd J, Brunton G, Oakley A (2004). Applying systematic review methods to studies of people's views: an example from public health research. J Epidemiol Community Health.

[B10] Barbour RS, Barbour M (2003). Evaluating and synthesising qualitative research: the need to develop a distinctive approach. J Eval Clin Pract.

[B11] Barbour RS (2001). Checklists for improving rigour in qualitative research: a case of the tail wagging the dog?. BMJ.

[B12] Noblit GW (1988). Meta-ethnography: synthesizing qualitative studies.

[B13] Campbell R, Pound P, Pope C, Britten N, Pill R, Morgan M, Donovan J (2003). Evaluating meta-ethnography: a synthesis of qualitative research on lay experiences of diabetes and diabetes care. Soc Sci Med.

[B14] Pound P, Britten N, Morgan M, Yardley L, Pope C, Daker-White G, Campbell R (2005). Resisting medicines: a synthesis of qualitative studies of medicine taking. Soc Sci Med.

[B15] Dixon-Woods M, Shaw RL, Agarwal S, Smith JA (2004). The problem of appraising qualitative research. Qual Saf Health Care.

[B16] Volmink J, Garner P (2007). Directly observed therapy for treating tuberculosis. Cochrane Database Syst Rev.

[B17] Munro S (2007). Patient adherence to tuberculosis treatment: a systematic review of qualitative research. PLoS Medicine.

[B18] Walsh D (2005). Meta-synthesis method for qualitative research: a literature review. Journal of Advanced Nursing.

[B19] Dixon-Woods M, Fitzpatrick R, Roberts K (2000). Including qualitative research in systematic reviews: opportunities and problems. J Eval Clin Pract.

[B20] Evans D (2002). Database searches for qualitative research. Journal of the Medical Library Association.

[B21] Barroso J (2003). The challenges of searching for and retrieving qualitative studies. Western Journal of Nursing Research.

[B22] Booth A (2001). Cochrane or cock-eyed? How should we conduct systematic reviews of qualitative research?. Qualitative Evidence-based conference: Taking a critical stance.

[B23] Shaw RL (2004). Finding qualitative research: an evaluation of search strategies. BMC Medical Research Methodology.

[B24] Spencer L (2003). Quality in qualitative evaluation: a framework for assessing research evidence. Government Chief Social Researcher’s Office Occasional Papers Series.

[B25] Malterud K (2001). Qualitative research: standards, challenges and guidelines. Lancet.

[B26] Rowan M (1997). Qualitative research articles: information for authors and peer reviewers. Canadian Medical Association Journal.

[B27] Programme CAS Critical Appraisal Skills Programme. Ten questions to help you make
sense of qualitative research. http://www.phru.nhs.uk/Doc_Links/Qualitative%20Appraisal%20Tool.pdf.

[B28] Sandelowski M, Barroso J (2002). Reading qualitative studies. International Journal of Qualitative Methods.

[B29] Anfara Jnr V (2002). Qualitative Analysis on Stage: Making the Research Process More Public. Educational Researcher.

[B30] Schutz A (1971). Collected Papers.

[B31] Smith LK (2005). Patient’s help seeking and delay in cancer presentation: a qualitative synthesis. Lancet.

[B32] Ritchie J (2003). Qualitative Health Research.

[B33] Walter FM (2004). Lay understanding of familial risk of common chronic diseases: a systematic review and synthesis of qualitative research. Annals of Family Medicine.

[B34] Garner P (2007). Promoting adherence to tuberculosis treatment. Bulletin of the World Health Organization.

[B35] Volmink J (1997). Systematic review of randomised controlled trials of strategies to promote adherence to tuberculosis treatment. BMJ.

[B36] Noyes J (2007). Directly observed therapy and tuberculosis: how can a systematic review of qualitative research contribute to improving services? A qualitative meta-synthesis. Journal of Advanced Nursing.

[B37] Baum F (1995). Researching public health: behind the qualitative-quantitative methodological debate. Social Science and Medicine.

